# The potential for prazosin and calcitonin gene-related peptide (CGRP) in causing hypoxia in tumours.

**DOI:** 10.1038/bjc.1991.381

**Published:** 1991-10

**Authors:** I. A. Burney, R. J. Maxwell, J. R. Griffiths, S. B. Field

**Affiliations:** Medical Research Council, Hammersmith Hospital, London, UK.

## Abstract

Using 31P NMR spectroscopy, changes in tumour metabolic status were studied in a transplanted rat fibrosarcoma following the administration of vasodilators. Mean Arterial Blood Pressure (MABP) was monitored simultaneously. Two vasodilators were studied, prazosin and CGRP, which altered the NMR parameters Pi/sigma P, beta NTP,Pi, PCr/Pi and PME/Pi in a dose dependent manner. There was a good correlation between the various NMR parameters; for analysis, Pi/sigma P was used for convenience. With increasing doses of vasodilator, Pi/sigma P increased and the MABP decreased. Reduction in pHNMR showed a correlation with decreasing MABP following the administration of prazosin but not after CGRP. Both prazosin and CGRP produced changes in 31P NMR spectra consistent with a reduction in tumour blood flow. The results for prazosin and CGRP were comparable and showed a 15-20% increase in Pi/sigma P for a 20% reduction in MABP. These results were compared with those from hydralazine. With hydralazine an acceptable reduction in blood pressure (up to approximately 25%) has little effect and may even alter NMR parameters consistent with an increase in blood flow, a reduction of approximately 40% is required for a significant decrease in flow. Both prazosin and CGRP are shown to be far more effective than hydralazine in causing tumour hypoxia at a clinically acceptable reduction in blood pressure. CGRP may be the more suitable for clinical use because of its short half life, its capability to achieve controlled hypotension and the relatively few side effects associated with its use.


Br. J. Cancer (1991), 64, 683-688 © Macmillan Press Ltd., 1991

### The potential for prazosin and calcitonin gene-related peptide (CGRP) in causing hypoxia in tumours

I.A. Burney[1], R.J. Maxwell[2], J.R. Griffiths[2] & S.B. Field[1]

[1]*Medical Research Council, Cyclotron Unit, Hammersmith Hospital, Ducane Road, London W12 0HS; and* [2]*CRC Biomedical Magnetic Resonance Research Group, Division of Biochemistry, Department of Cellular and Molecular Sciences, St George's Hospital and Medical School, London SW17 0RE, UK.*

**Summary** Using $^{31}P$ NMR spectroscopy, changes in tumour metabolic status were studied in a transplanted rat fibrosarcoma following the administration of vasodilators. Mean Arterial Blood Pressure (MABP) was monitored simultaneously. Two vasodilators were studied, prazosin and CGRP, which altered the NMR parameters Pi/ΣP, βNTP,Pi, PCr/Pi and PME/Pi in a dose dependent manner. There was a good correlation between the various NMR papameters; for analysis, Pi/ΣP was used for convenience. With increasing doses of vasodilator, Pi/ΣP increased and the MABP decreased. Reduction in $pH_{NMR}$ showed a correlation with decreasing MABP following the administration of prazosin but not after CGRP.

Both prazosin and CGRP produced changes in $^{31}P$ NMR spectra consistent with a reduction in tumour blood flow. The results for prazosin and CGRP were comparable and showed a 15–20% increase in Pi/ΣP for a 20% reduction in MABP. These results were compared with those from hydralazine. With hydralazine an acceptable reduction in blood pressure (up to ≈25%) has little effect and may even alter NMR parameters consistent with an *increase* in blood flow, a reduction of ≈40% is required for a significant decrease in flow.

Both prazosin and CGRP are shown to be far more effective than hydralazine in causing tumour hypoxia at a clinically acceptable reduction in blood pressure. CGRP may be the more suitable for clinical use because of its short half life, its capability to achieve controlled hypotension and the relatively few side effects associated with its use.

Selective reduction in tumour blood flow and the consequent increase in hypoxia could have several advantages in cancer treatment, for example in combination with drugs which are selectively toxic to hypoxic cells (Brown, 1987; Chaplin & Acker, 1987; Stratford *et al.*, 1989; Bremner *et al.*, 1990). It might also be an advantage in treatment by hyperthermia, resulting in higher tumour temperatures (Babbs *et al.*, 1982) and more uniform heating together with increased tumour sensitivity resulting from decreased nutrient delivery and reduced pH (Gerweck *et al.*, 1979; Horsman *et al.*, 1989).

The potential for selective manipulation of tumour blood flow depends on differences in vasculature between tumours and normal tissues. Tumours are thought to have long, tortuous, leaky vessels with frequent A-V shunts, lack of smooth muscle and probably no innervation. Interstitial pressure tends to be high due to leaky vessels (Wiig *et al.*, 1982) and lack of lymph drainage (see Reinhold & Endrich, 1986 for review). The response of the vasculature of a tumour to a vasodilator is likely to be different from that of a normal tissue for two reasons (Chan *et al.*, 1984). One is that the tumour arterioles may react minimally or not at all to the vasoactive stimuli so that vasodilation elsewhere results in 'stealing' of blood from the tumour. The second results from the normally high tumour interstitial fluid pressure so that any reduction in capillary hydrostatic pressure may lead to tumour vascular collapse leading to flow stasis or even reversal (Chaplin *et al.*, 1987; Trotter *et al.*, 1988; Trotter *et al.*, 1989).

The most successful attempts to reduce tumour blood flow has been by the administration of hydralazine, a directly acting vasodilator (Babbs *et al.*, 1982). This has been shown to decrease tumour blood flow in experimental animals and also to potentiate the action of bioreductive compounds and hyperthermia (Brown, 1987; Chaplin *et al.*, 1987; Horsman *et al.*, 1989). However, to achieve these effects with hydralazine, a substantial reduction in blood pressure is required, which is likely to limit the clinical use of hydralazine as a modifier of tumour blood flow (Okunieff *et al.*, 1989; Tozer *et al.*, 1990). For a reasonable, clinically acceptable reduction in blood pressure, hydralazine has even been shown to cause an *increase* in tumour blood flow (Kalmus *et al.*, 1990; Rowell *et al.*, 1990). A further disadvantage of hydralazine is its long half life (Gross, 1977). Clearly, there is a need to investigate drugs which could be of greater therapeutic benefit.

Information related to tumour oxygenation and perfusion status can be obtained from changes in bioenergetic status using $^{31}P$ Magnetic Resonance Spectroscopy (Evelhoch *et al.*, 1986; Rofstad *et al.*, 1989). This technique has been used as a non-invasive means of monitoring tumour metabolic status following the administration of vasodilators hydralazine and prostacyclin (Okunieff *et al.*, 1989; Bhujwalla *et al.*, 1990*a*; Tozer *et al.*, 1990). Recently a direct relationship between tumour metabolic status and blood flow has been demonstrated (Bhujwalla *et al.*, 1990*b*). In the present study, we have used $^{31}P$ MRS to obtain information on tumour blood flow by monitoring changes in tumour metabolism following administration of various vasoactive compounds. Blood pressure was monitored simultaneously, since this falls substantially following administration of vasodilators. The results from two vasodilators, prazosin and CGRF, are reported. Prazosin is a quinazoline derivative which acts by competitive blockade of post-synaptic $\alpha_1$-adrenergic receptors. Calcitonin gene-related peptide (CGRP) is an endogenously occurring peptide (Rosenfeld *et al.*, 1983; Morris *et al.*, 1984), which acts by binding to its receptors in the vessel wall (Lundberg *et al.*, 1985). When given intravenously CGRP is one of the most potent vasodilators known (Brain *et al.*, 1985; Struthers *et al.*, 1986). It has the advantage of a short half life (Benjamin *et al.*, 1987). The mechanism of action of each of these compounds is different from that of hydralazine, which acts by directly relaxing the arteriolar smooth muscle.

### Materials and methods

### *Tumour model*

A transplanted rat fibrosarcoma, designated $LBDS_1$, was used for these experiments. The tumour arose spontaneously

Correspondence: I.A. Burney.
Received 13 February 1991; and in revised form 21 May 1991.

in the flank of a male $BD_9$ rat and was serially transplanted. Further details of these tumours may be found elsewhere (Tozer & Morris, 1990). Only early generation transplants were used. Tumour pieces $1-2\,mm^3$, taken from a previous generation isotransplant were implanted subcutaneously into the right flanks of 10–12 week old $BD_9$ rats. The rats were then returned to their cages and housed in a temperature controlled and light-cycled room. They were fed on normal rat chow and water *ad libitum*. It took an average time of $30 \pm 4$ days for the tumours to reach 15–20 mm in diameter, at which size they were used for experiments.

### *Drugs used*

In order to restrain the animals for NMR spectroscopy, they were anaesthetised with a combination of fentanyl citrate ($0.315\,mg\,kg^{-1}$), fluanisone ($10\,mg\,kg^{-1}$) 'Hypnorm' (Crown Chemical Co.) and midazolam ($5\,mg\,kg^{-1}$) 'Hypnovel' (Roche).

Prazosin (Sigma Chemical Co) was freshly dissolved in water to give an iso-volume of 0.5 ml for various doses and injected intravenously in bolus doses of 0.5, 1.0 and 1.5 mg $kg^{-1}$ rat body weight.

Rat calcitonin gene-related peptide 'CGRP' (Peninsula Lab) was dissolved in distilled, deionised water containing 0.001% (v/v) acetic acid and 0.1% (w/v) bovine serum albumin, and stored at −70°C (Zaidi *et al.*, 1989). For intravenous injection, the mixture was re-dissolved in saline, to give a constant injection volume of 0.5 ml for various doses. CGRP was injected in bolus doses of 300 pmol ($5.8\,\mu g\,kg^{-1}$), 600 pmol ($10.8\,\mu g\,kg^{-1}$), 1 nmol ($20.2\,\mu g\,kg^{-1}$) and 2 nmol ($38.8\,\mu g\,kg^{-1}$).

### *Spectroscopy*

$^{31}P$ spectra were obtained on a 1.89T Oxford Research Systems TMR-32 spectrometer. A range of radiofrequency surface coils was used, the size (1–2 cm diameter) being chosen to fit closely around the tumour. The magnetic field was shimmed to obtain a $H_2O$ resonance with a maximum linewidth of 40 Hz. Experimental parameters included a spectral width of 2 KHz, 90° pulse with a pulse length of 10 µs, pulse repetition time of 2 s, 2048 data points and 300 or 600 averaged free induction decays. Data processing involved exponential line broadening of 15 Hz. Broad spectral lines were removed by spectral deconvolution (Tozer *et al.*, 1989). Peak areas were calculated by a computer program which allowed operator definition of the baseline and peak limits. No assumptions were made about the peak shapes in the analysis of spectra. However there could be a systematic error from small signals due to peak overlap. This error would be significant only if the area of the peak were considerably different from that of neighbouring peaks. For example if the area the inorganic phosphate peak were 2-fold greater than that of the neighbouring peaks, it would be underestimated by approximately 10%. Consequently changes observed in the spectra following treatment with vasodilators would be slightly underestimated. Non-systematic error in estimating the peak area would have been of the order of 10%, given the signal-to-noise ratio obtained in the collection of spectra.

### *Blood pressure measurements*

Mean arterial blood pressure was monitored using a Gould P23XL physiological pressure transducer connected to a Gould RS3200 recorder (see below).

### *Experimental protocol*

The rats were anaesthetised and polythene catheters containing heparinised normal saline were implanted into a tail vein and tail artery. The tail artery catheter was connected to the pressure transducer by a sufficient length of pressure tubing to monitor the changes whilst the rat was within the bore of the magnet. The rat was placed on two plastic tissue culture flasks containing recirculating warm water to maintain core temperature at 37°C. It was positioned so that its tumour hung vertically downwards between the two culture flasks and rested on the surface coil. A baseline spectrum was accumulated over a period of 20 min, following which either prazosin or CGRP was injected via the tail vein catheter without disturbing the position of the rat in the magnet. Control rats were injected with 0.5 ml of saline. Spectra were accumulated for at least 80 min following prazosin or water and for up to 40 min following CGRP. Blood pressure was monitored throughout.

### *Data analysis*

The results given in both the text and figures are means and standard errors for the given number of rats.

### Results

A typical $^{31}P$ NMR spectrum from a tumour is shown in Figure 1a together with sequential spectra following an intravenous bolus injection of 600 pmol CGRP. The MABP recorded simultaneously is shown in Figure 1b. The principal change in NMR spectrum was an increase in the amplitude of the Pi peak, which was maximal in the first 10 min period, whilst the blood pressure was at its minimum. With CGRP there was a fairly rapid recovery of blood pressure, with Pi, PCr and NTP peaks returning to control values more slowly. Following prazosin, the changes in MRS parameters and reduction in MABP were not reversed during the 80 min period of study, consistent with the long half life of this drug.

With both prazosin and CGRP, the reduction in MABP and corresponding increase in Pi/ΣP were dose dependent. Thus an inverse relationship was found between decreasing MABP and increasing Pi/ΣP with escalating doses of vasodilator, as shown in Figure 2 for CGRP. A similar result was found for prazosin.

NMR parameters other than Pi/ΣP, i.e. βNTP/Pi, PCr/Pi and PME/Pi were also recorded. βNTP/Pi and PCr/Pi showed a good correlation with Pi/ΣP as shown in Figure 3a and b. Tables I and II show that the parameters Pi/ΣP, βNTP/Pi, PCr/Pi and PME/Pi were all correspondingly altered as a

**Figure 1** **A**, Sequential $^{31}P$ NMR spectra from a rat fibrosarcoma following the administration of 600 pmol CGRP. **a**, before drug; **b**, 5 min; **c**, 15 min; **d**, 25 min after the injection of CGRP. PME = Phosphomonoesters; Pi = Inorganic phosphate; PDE = Phophodiesters; PCr = Phosphocreatine; γNTP = γ nucleotide triphosphate; αNTP = α nucleotide triphosphate; βNTP = β nucleotide triphosphate. **B**, A simultaneously recorded trace of MABP from the same study. Dark vertical line represents the time of injection of the drug.

Figure 2 Pi/ΣP and MABP followed as a function of dose of CGRP. Each point represents mean for three animals. —□— Pi/ΣP, —◆— MABP.

Figure 3 A, Correlation between NMR parameters Pi/ΣP and PCr/Pi following injection of two vasodilators. Each point represents a mean from three animals. $r = 0.66$. □, Prazosin; ◆, CGRP. B, Correlation between NMR parameters Pi/ΣP and βNTP/Pi following injection of two vasodilators. Each point represents a mean from three animals. $r = 0.44$. □, Prazosin; ◆, CGRP.

function of dose of prazosin or CGRP. No significant change was observed in these parameters in control animals treated with saline. Since these parameters are all interdependent, it was decided to concentrate the analysis of Pi/ΣP for convenience.

The time course of changes of Pi/ΣP and blood pressure following an injection of 1 mg $kg^{-1}$ prazosin is shown in Figure 4. It is seen that following injection, there was a

**Table I** Effects of different doses of prazosin on NMR parameters

| *Dose* | *Pi/ΣP* | *βNTP/Pi* | *PCr/Pi* | *PME/Pi* |
|---|---|---|---|---|
| Control | 102±13 | 92±12 | 95±10 | 114±09 |
| 0.5 mg $kg^{-1}$ | 123±02 | 71±12 | 77±08 | 87±11 |
| 1.0 mg $kg^{-1}$ | 148±09 | 60±13 | 65±10 | 85±16 |
| 1.5 mg $kg^{-1}$ | 167±25 | 56±07 | 57±06 | 87±03 |

Figure are percentages. Values are from the data collected 50 min after the administration of prazosin. Results are mean±s.e.m. from three animals in each group.

**Table II** Effects of different doses of CGRP on NMR parameters

| *Dose* | *Pi/ΣP* | *βNTP/Pi* | *PCr/Pi* | *PME/Pi* |
|---|---|---|---|---|
| Control | 106±07 | 93±09 | 101±12 | 104±05 |
| 300 pmol | 116±14 | 88±14 | 102±22 | 88±14 |
| 600 pmol | 126±06 | 79±05 | 78±00 | 80±07 |
| 1 nmol | 140±09 | 66±13 | 56±11 | 69±05 |
| 2 nmol | 142±17 | 48±03 | 57±07 | 79±14 |

Figures are percentages. Values are from the data collected 5 or 10 min after the administration of CGRP. Results are mean±s.e.m. from three animals in each group.

Figure 4 Per cent change in Pi/ΣP and MABP as a function of time following a dose of prazosin of 1 mg $kg^{-1}$. Blood pressure is averaged over a period of 20 min. Each point represents mean±s.e.m. from three animals. —□—, Pi/ΣP; —◆—, MABP.

steady fall in MABP with a corresponding increase in Pi/ΣP. At 70 min both were still changing. The response of Pi/ΣP and MABP following administration of a bolus dose of 2 nmol CGRP is shown in Figure 5. In this case it can be seen that there was a rapid fall in MABP with a corresponding rapid increase in Pi/ΣP. However, the return towards normal of MABP began after a few minutes, whereas Pi/ΣP recovered more slowly. In all these studies the rats were anaesthetised by a combination of hypnorm and hypnovel. This anaesthetic slightly reduces the blood pressure (Field & Burney, unpublished). However, any interactions between the vasodilators and the anaesthetic are not known.

### Discussion

There is a strong evidence that $^{31}P$ MRS can detect changes in tumour bioenergetics brought about by changes in tumour oxygenation and perfusion. For example, Evelhoch *et al.* (1986), using *in situ* photon activation-$^{15}O$ decay measurements and $^{31}P$ NMR spectroscopy, showed a positive correlation between $^{15}O$ perfusion and NTP/Pi and PCr/NTP ratios. A positive correlation was also observed between declining mean tissue $pO_2$ and NTP/Pi, PCr/Pi, PME/Pi, PDE/Pi and $pH_{RNMR}$ with increasing tumour size in a murine fibrosar-

**Figure 5** Per cent change in Pi/ΣP and MABP as a function of time following a dose of 2 nmol CGRP. Blood pressure is averaged over a period of 10 min. Each point represents mean ± s.e.m. from three animals. —□—, Pi/ΣP; —◆—, MABP.

**Figure 6** Per cent change in Pi/ΣP plotted as a function of per cent reduction in MABP. Each point represents mean from three or four animals. Blood pressure is averaged over a period of 10 min. Curve A is for prazosin and CGRP. Curve B is for hydralazine. The curves were drawn by eye. 100% Pi/ΣP is the reference point, anything above 100% implies a reduction in blood flow and anything below 100% implies an improvement. ■, Hydralazine; O, Prazosin; +, CGRP.

coma (Vaupel *et al.*, 1989). Rofstad *et al.* (1989) and Mueller-Klieser *et al.* (1990) have both reported a decreasing tumour bioenergetic status together with a decreasing $HbO_2$ saturation levels as measured by cryospectrophotometry. More recently Bhujwalla *et al.* (1990*b*) have also shown a direct correlation between βNTP/Pi and tumour blood flow.

The most effective vasodilator reported to cause hypoxia in experimental tumours appears to be hydralazine which acts directly on smooth muscle (Babbs, 1982; Voorhees & Babbs, 1982; Brown, 1987; Chaplin & Acker, 1987; Horsman *et al.*, 1989; Stratford *et al.*, 1989). The effect results either from diversion of blood away from the tumour to the normal tissues termed the 'steal phenomenon' or from the reduction in systemic blood pressure, leading to vascular collapse (Chaplin *et al.*, 1987; Trotter *et al.*, 1989). Tozer *et al.* (1990) used $^{31}P$ NMR to demonstrate that hydralazine is more effective than prostacyclin in causing changes consistent with reduction in tumour perfusion for a given reduction in blood pressure.

Hydralazine can cause almost complete radiobiological hypoxia in tumours but this requires high doses of drugs resulting in a massive and clinically unacceptable reduction in blood pressure (Okunieff *et al.*, 1989; Tozer *et al.*, 1990). At lower doses of hydralazine ($\approx$0.1 mg kg$^{-1}$ in rodents), resulting in a clinically acceptable reduction in blood pressure (up to $\approx$25%), laser doppler flow studies show that tumour blood flow may actually be improved (Kalmus *et al.*, 1990). Some NMR studies also show changes consistent with improved tumour blood flow following lower doses of hydralazine (Okunieff *et al.*, 1989; Tozer *et al.*, 1990). These experimental results are consistent with the clinical studies of Rowell *et al.* (1990) who showed an increase in tumour blood flow measured by Single Photon Emission Computed Tomography (SPECT) in patients given oral hydralazine.

Taken together these results show that at a clinically acceptable reduction in blood pressure, hydralazine may even cause an *improved* blood flow and only with a clinically unacceptably large reduction in blood pressure is blood flow reduced. In addition, hydralazine has a fairly long half life (Gross, 1977). Clearly hydralazine is unlikely to be of clinical use in reducing tumour blood flow and more effective vasodilators are needed for this purpose.

Prazosin and CGRP both produced changes in NMR spectra consistent with a reduction in tumour blood flow for much smaller reduction in blood pressure than with hydralazine. The relative effectiveness of these compounds is demonstrated in Figure 6, in which an injection resulting in a 20% drop in MABP, causes at least a 15% increase in Pi/ΣP. In contrast, a similar drop in blood pressure with hydralazine leads to a 15% *decrease* in Pi/ΣP, consistent with an increase in blood flow. With hydralazine it is necessary to give sufficient dose to cause at least a 30–40% reduction in blood pressure to achieve a significant increase in Pi/ΣP.

In control animals treated with saline, the variation in the NMR parameters was relatively small. As seen in Tables I and II, the variation in the ratios Pi/ΣP, βNTP/Pi, PCr/Pi and PME/Pi was of the order of 2–8%. The variability in the area of the same individual peak in spectra collected from the same control tumour ranged between 4–12%. The experimental design, such that the rat was not moved within the magnet throughout the course of the experiment, the drug being administered remotely, ensured that any change in spectrum was due solely to the administration of the drug.

The use of anaesthetics to immobilise the rats during the procedures is almost certainly less perturbing than the methods to forcibly restrain them. The anaesthetic used was a combination of a morphine type analgesic, fentanyl; a neuroleptic of butyrophenene group, fluanisone; and a benzodiazepine, midazolam; all mediating their effect through the centra nervous system. This combination anaesthetic was chosen because it has the advantage over other commonly used anaesthetics (such as pentobarbital, ketamine, morphine, halothane and enflurane), of not increasing the peripheral vascular resistance, conferring better stress protection and preserving tissue perfusion (Skolleborg *et al.*, 1990). Although no interaction between this combination anaesthetic and the different vasodilators used in this study has been documented, it seems unlikely that any such interaction would occur between such centrally acting anaesthetic agents and the tested vasoactive compounds which mediate their effect through specific receptors. Furthermore, this combination anaesthetic has little or no effect on the release of corticosteroids, which play an important role in central haemodynamics and an interaction with the hypothalamic-hypophyseal-cortical axis also seems unlikely.

Whereas, midazolam has little or no effect on various haemodynamic parameters, fentanyl and fluanisone do decrease the mean aortic blood pressure and increase the cardiac output, although the reduction in blood pressure is less than that produced by the other commonly used anaesthetics e.g. pentobarbitone, ketamine or diazepam (Cullen & Walker, 1986). The aim of the study was to relate changes in NMR parameters to changes in blood pressure. If there were any effect of the anaesthetic it would almost certainly be confined to causing a small additional reduction in blood pressure and would be a common factor throughout, including all similar studies with hydralazine, which have been used for comparison to the effects of CGRP and prazosin. Any effect of the anaesthetic would therefore not alter the conclusions.

**Table III** Plasma and biological half-lives of three vasodilators in man

| Drug | Half-life in man Plasma | Biological |
|---|---|---|
| Hydralazine | 4 h | 30 h |
| Prazosin | 2–3 h | 10–12 h |
| CGRP | 9 min | 19 min |

Prazosin may not be suitable for clinical use as a modifier of tumour blood flow because of its long half life (see Table III). The hypotensive effects may last for up to 10–12 h (Stanaszek *et al.*, 1983). Faintness and dizziness have been reported to occur in 50% of patients receiving this drug. Severe orthostatic hypotension is less common and is attributed to the 'first dose effect' (Rudd & Blaschke, 1985).

One of the limitations for the clinical use of CGRP might be the sudden reduction in blood pressure during the first few minutes following the injection of a bolus dose. This may be particularly harmful to elderly patients. However, it has been shown that CGRP can be administered safely by infusion to achieve controlled hypotension (Struthers *et al.*, 1986). CGRP has a short plasma and biological half life in man (see Table III). This may be a very effective method of administration since the effects on metabolism are more prolonged than the reduction in blood pressure (Figure 5). Lack of tachyphylaxis and other side effects associated with prazosin and hydralazine, make CGRP the more favourable for clinical use.

The increase in tumour blood flow following low doses of hydralazine suggested by a decrease in Pi/ΣP and by other techniques (Kalmus *et al.*, 1990; Rowell *et al.*, 1990) can be explained by increased perfusion pressure occurring as a result of reflex sympathetic stimulation in the presence of little vasodilatation (Maekawa *et al.*, 1984). Prazosin blocks the action of nor-epinephrine by post-synaptic receptor blockade and subsequently decreases its further release through negative feed back control (Stanaszek *et al.*, 1983). CGRP causes sympathetic stimulation (Fisher *et al.*, 1983), increasing the nor-epinephrine release by almost 2-fold. Although the mechanism of action of these drugs is far from fully understood, there appears to be a similarity in the mode of action of prazosin and CGRP, both being receptor mediated, in contrast to hydralazine which is known to cause arteriolar vasodilatation by direct relaxation of smooth muscle (Åblad, 1963). Whether this difference in the mode of action is the cause of prazosin and CGRP being substantially more effective than hydralazine in modifying tumour blood flow for a given reduction in MABP, remains speculative. It is plausible however, that compounds with a receptor mediated mode of action, because of sensitive vasomotor control, preferentially cause vascular collapse and flow stasis, thus rendering tumours hypoxic for small reduction in blood pressure. In contrast, diversion of blood by the 'steal' phenomenon to cause hypoxia probably requires a greater reduction in systemic blood pressure.

In conclusion, two vasodilators, prazosin and CGRP, have been shown to produce changes in MRS parameters consistent with a reduction in tumour blood flow at a substantially smaller reduction in mean arterial blood pressure than hydralazine. CGRP has the greater potential for clinical use because it has a short half life and a lower probability of causing side effects. Studies are in progress using PET to monitor changes in human tumour blood flow following administration of CGRP. There is however a need to understand better the mechanism of action of these drugs in the context of pathophysiology of tumour vasculature, if they are to be used to maximal effect.

This work was supported by a grant from Medical Research Council and a grant from Cancer Research Campaign.

### References

ÅBLAD, B. (1963). Study of the mechanism of the haemodynamic effects of hydralazine in man. *Acta Pharmacol. Toxicol.*, **20**, 1.

BABBS, F., DEWITT, D.P., VORHEES, W.D., MCCAW, J.S. & CHAN, R.C. (1982). Theoretical feasibility of vasodilator enhanced local tumour heating. *Eur. J. Cancer Clin. Oncol.*, **18**, 1137.

BENJAMIN, N., DOLLERY, C.T., FULLER, R.W., LARKINS, S. & MCEWAN, J. (1987). The effects of calcitonin-gene related peptide and substance P on resistance and capacitance vessels. *Br. J. Pharmacol.*, **90** (Suppl), 43.

BHUJWALLA, Z.M., TOZER, G.M., FIELD, S.B., MAXWELL, R.J. & GRIFFITHS, J.R. (1990*a*). The energy metabolism of RIF-1 tumours following hydralazine. *Radiother. & Oncol.*, **19**, 281.

BHUJWALLA, Z.M., TOZER, G.M., FIELD, S.B., PROCTOR, E., BUSZA, A. & WILLIAMS, S.R. (1990*b*). The combined measurements of blood flow and metabolism in RIF-1 tumours *in vivo*. A study using $H_2$ flow and $^{31}P$ NMR spectroscopy. *NMR Biomed.*, **3**, 178.

BRAIN, S.D., WILLIAMS, T.J., TIPPINS, J.R., MORRIS, H.R. & MCINTYRE, I. (1985). Calcitonin gene-related peptide is a potent vasodilator. *Nature*, **313**, 54.

BREMNER, J.C.M., STRATFORD, I.J., BOWLER, J. & ADAMS, G.E. (1990). Bioreductive drugs and the selective induction of tumour hypoxia. *Br. J. Cancer*, **61**, 717.

BROWN, J.M. (1987). Exploitation of bioreductive agents with vasoactive drugs. In *Radiation Research*, Vol 2, Fielden, E.M., Fowler, J.F., Hendry, J.H. & Scott, D. (eds). pp. 719–724. Taylor & Francis: London.

CHAPLIN, D.J. & ACKER, B. (1987). The effect of hydralazine on the tumour cytotoxicity of hypoxic cell cytotoxin RSU-1069: evidence for therapeutic gain. *Int. J. Radiat. Oncol. Biol. Phys.*, **13**, 579.

CHAPLIN, D.J., OLIVE, P. & DURAND, R.E. (1987). Intermittent blood flow in a murine tumor: radiobiological effects. *Cancer Res.*, **13**, 579.

CHAN, R.C., BABBS, C.F., VETTER, R.J. & LAMAR, C.H. (1984). Abnormal response of tumour vasculature to vasoactive drugs. *J. Natl Cancer Inst.*, **72**, 145.

CULLEN, B.M. & WALKER, H.C. (1986). The effect of several different anaesthetics on the blood pressure and heart rate of the mouse and on the radiation response of the mouse sarcoma RIF-1. *Int. J. Radiat. Biol.*, **48**, 761.

EVELHOCH, J.L., SAPARETO, S.A., NUSSBAUM, G.H. & ACKERMAN, J.J.H. (1986). Correlations between $^{31}P$ NMR spectroscopy and $^{15}O$ perfusion measurements in RIF-1 murine tumour *in vivo*. *Radiat. Res.*, **106**, 122.

FISHER, L.A., KIKKAWA, D.D., RIVIER, J.A. & 5 others (1983). Stimulation of noradrenergic sympathetic outflow by calcitonin-gene related peptide. *Nature*, **305**, 534.

GERWECK, L.E., TOBJOERN, G.N. & BURLETT, M. (1979). Response of cells to hyperthermia under acute and chronic hypoxic conditions. *Cancer Res.*, **39**, 966.

GROSS, F. (1977). Drug acting on arteriolar smooth muscle (Vasodilator drugs). Gross, F. (ed.). *Anti-Hypertensive Agents*. p. 397. Springer-Verlag: Berlin.

HORSMAN, M.R., CHRISTENSEN, K.L. & OVERGAARD, J. (1989). Hydralazine induced enhancement of hyperthermic damage in a C3H mammary carcinoma *in vivo*. *Int. J. Hyperthermia*, **5**, 123.

KALMUS, J., OKUNIEFF, P. & VAUPEL, P. (1990). Dose-dependent effects of hydralazine on microcirculatory function and hyperthermic response of murine FSaII tumours. *Cancer Res.*, **50**, 15.

LUNDBERG, J.M., FRANCO-CERECEDA, A., HUA, Y., HOKFELT, T. & FISCHER, J.A. (1985). Co-existence of substance P and calcitonin-gene related peptide-like immunoreactivities in sensory nerves in relation to cardiovascular and broncho-constrictor effects of capsaicin. *Eur. J. Pharmacol.*, **108**, 315.

MAEKAWA, K., CHANG-SENG, L., TSUI, A., CHEN, B.T. & KAWASHIMA, S. (1984). Vasodilative effect of hydralazine in awake dogs: the role of prostaglandins and sympathetic nervous system. *Circulation*, **70**, 908.

MORRIS, H.R., PANICO, M., ETIENNE, T., TIPPINS, J., GIRGIS, S.I. & MACINTYRE, I. (1984). Isolation and characterization of human calcitonin gene-related peptide. *Nature*, **308**, 746.

MULLER-KLIESER, W., SCHAEFER, C., WALENTA, S., ROFSTAD, E.K., FENTON, B.M. & SUTHERLAND, R.M. (1990). Assessment of tumour energy and oxygenation status by bioluminescence, nuclear magnetic resonance spectroscopy, and cryospectrophotometry. *Cancer Res.*, **50**, 1681.

OKUNIEFF, P., WALSH, C.S., VAUPEL, P. & 4 others (1989). Effects of hydralazine on *in vivo* tumour energy metabolism, Haematopoetic radiation sensitivity, and cardiovascular parameters. *Int. J. Radiat. Oncol. Biol. Phys.*, **16**, 1145.

REINHOLD, H.S. & ENDRICH, B. (1986). Tumour microcirculation as a target for hyperthermia. *Int. J. Hyperthermia*, **2**, 111.

ROFSTAD, E.K., DEMUTH, P., FENTON, B.M., CECKLER, T.L. & SUTHERLAND, R.M. (1989). $^{31}P$ NMR spectroscopy and $HbO_2$ cryospectrophotometry in prediction of tumour radioresistance caused by hypoxia. *Int. J. Radiat. Oncol. Biol. Phys.*, **16**, 919.

ROSENFELD, M.G., MERMOD, J.J., AMARA, S.G. & 5 others (1983). Production of a novel neuropeptide by calcitonin gene via tissue-specific RNA processing. *Nature*, **304**, 129.

ROWELL, N.P., FLOWER, M.A., MCCREADY, V.R., CRONIN, B. & HORWICH, A. (1990). The effects of single dose oral hydralazine on blood flow through human lung tumour. *Radiother. & Oncol.*, **18**, 283.

RUDD, P. & BLASCHKE, T.F. (1985). Antihypertensive agents and the drug therapy of hypertension. In *The Pharmacological Basis of Therapeutics*. Goodman, L.S. & Gillman, A. (eds), p. 793. Macmillan: New York.

SKOLLEBORG, K.C., GRONBECH, J.E., GRONG, K., ABYHOLM, F.E. & LEKVEN, J. (1990). Distribution of cardiac output during pentobarbital versus midazolam/fentanyl/fluanisone anaesthesia in the rat. *Lab. Anim.*, **24**, 221.

STANASZEK, W.F., KELLERMAN, D., BROGDEN, R.N. & ROMANKIEWICZ, J.A. (1983). Prazosin update: a review of its pharmacological properties and therapeutic use in hypertension and congestive heart failure. *Drugs*, **25**, 339.

STRATFORD, I.J., ADAMS, G.E., GODDEN, J. & HOWELLS, N. (1989). Induction of tumour hypoxia post-irradiation: a method for increasing the sensitizing efficiency of misonidazole and RSU-1069 *in vivo*. *Int. J. Radiat. Biol.*, **55**, 411.

STRUTHERS, A.D., BROWN, M.J., MACDONALD, D.W.R. & 4 others (1986). Human calcitonin-gene related peptide: a potent endogenous vasodilator in man. *Clin. Sci.*, **70**, 389.

TOZER, G.M., BHUJWALLA, Z.M., GRIFFITHS, J.R. & MAXWELL, R.J. (1989). Phosphorous-31 magnetic resonance spectroscopy and blood perfusion of the RIF-1 tumour following X-irradiation. *Int. J. Radiat. Oncol. Biol. Phys.*, **16**, 155.

TOZER, G.M. & MORRIS, C.C. (1990). Blood flow and blood volume in a transplanted rat fibrosarcoma: comparison of various normal tissues. *Radiother. & Oncol.*, **17**, 153.

TOZER, G.M., MAXWELL, R.J., GRIFFITHS, J.R. & PHAM, P. (1990). Modification of the $^{31}P$ magnetic resonance spectra of a rat tumour using vasodilators and its relation to hypotension. *Br. J. Cancer*, **62**, 553.

TROTTER, M.J., ACKER, B. & CHAPLIN, D.J. (1988). Hydralazine induced vascular collapse in murine tumours. *Int. J. Radiat. Oncol. Biol. Phys.*, **15**, 128.

TROTTER, M.J., ACKER, B. & CHAPLIN, D.J. (1989). Histological evidence for nonperfused vasculature in a murine tumour following hydralazine administration. *Int. J. Radiat. Oncol. Biol. Phys.*, **17**, 785.

VAUPEL, P., OKUNIEFF, P., KALLINOWSKI, F. & NEURINGER, L.J. (1989). Correlations between $^{31}P$ NMR spectroscopy and tissue $O_2$ tension measurements in a murine fibrosarcoma. *Radiat. Res.*, **20**, 477.

VORHEES, W.D. & BABBS, C.F. (1982). Hydralazine enhanced selective heating of transmissible venereal implants in dogs. *Eur. J. Cancer Clin. Oncol.*, **18**, 1027.

WIIG, H., TVIET, E., HULTBORN, R., REED, R.K. & WIESS, L. (1982). Interstitial fluid pressure in DMBA-induced rat mammary tumours. *Scand. J. Clin. Lab. Invest.*, **42**, 159.

ZAIDI, M., BREIMER, L.H. & MACINTYRE, I. (1989). Production of calcitonin-gene related peptide from human cancer cells. *J. Endocrinol.*, **123**, 159.


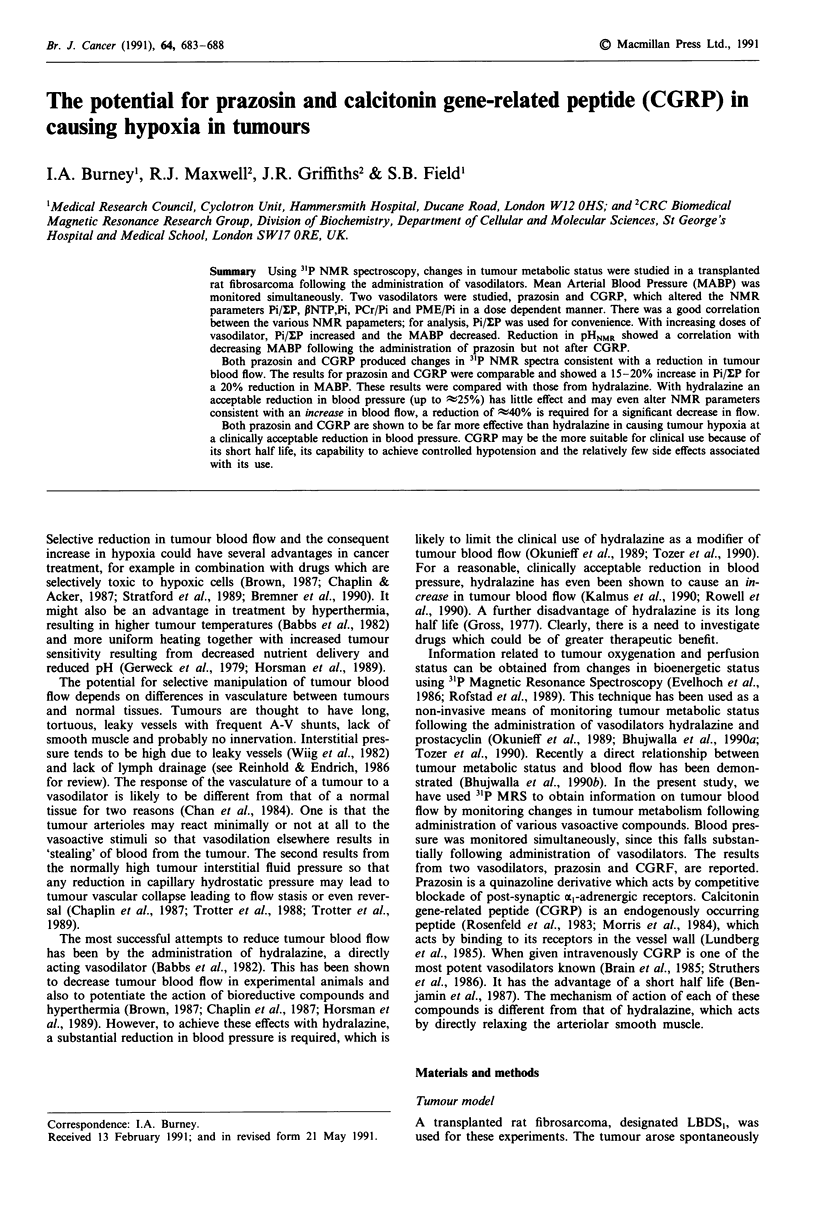

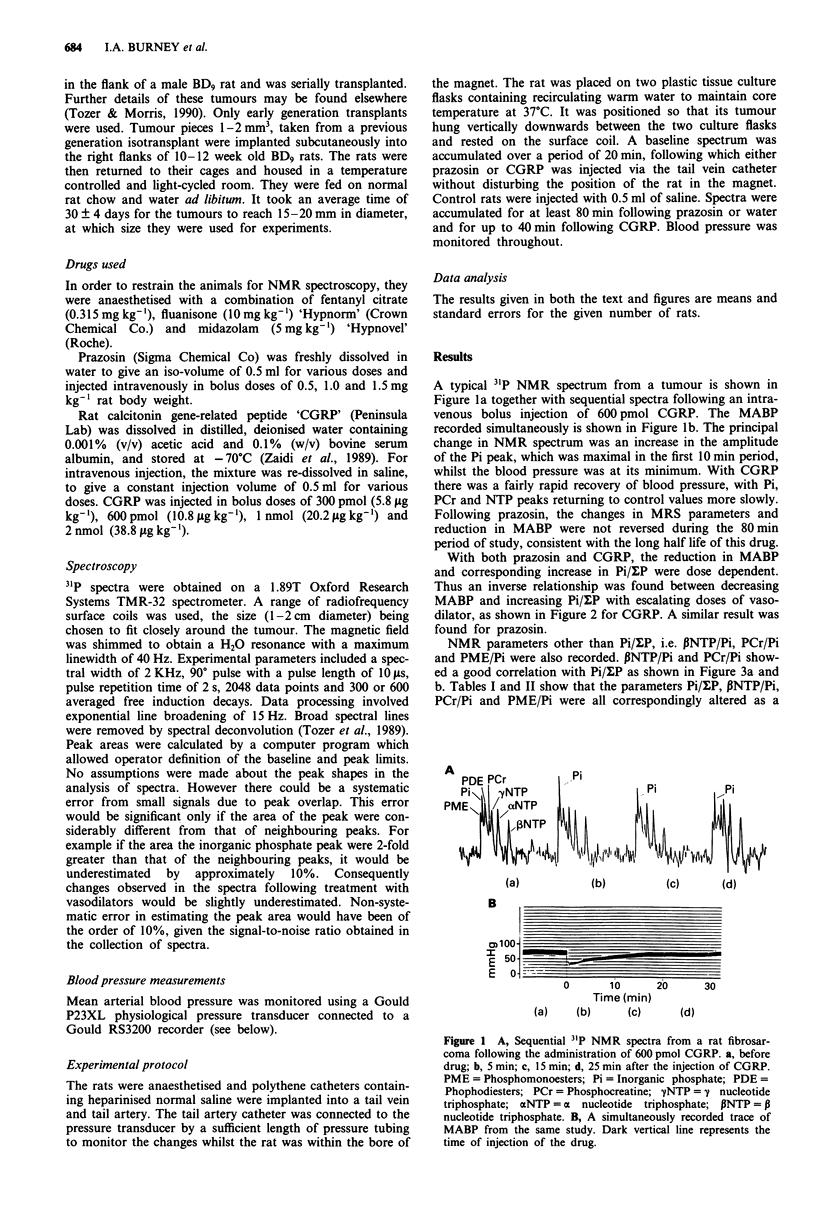

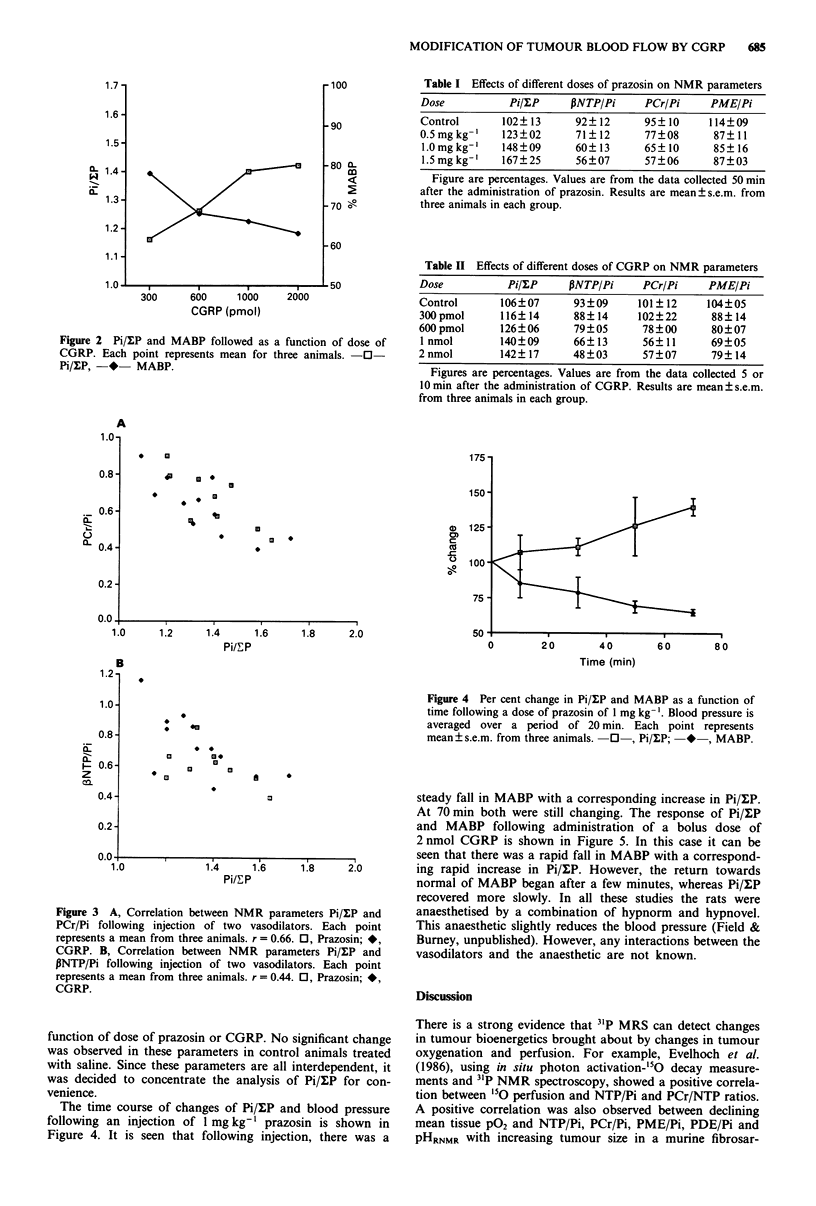

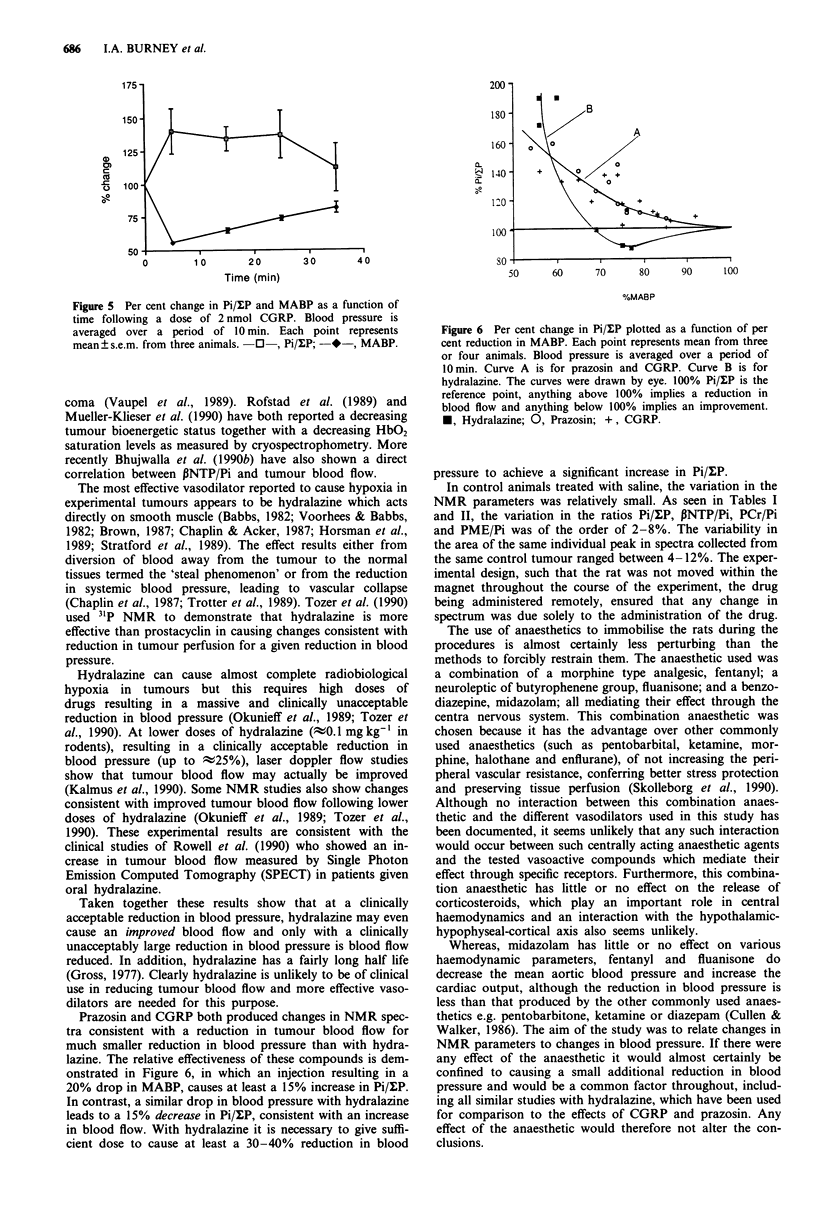

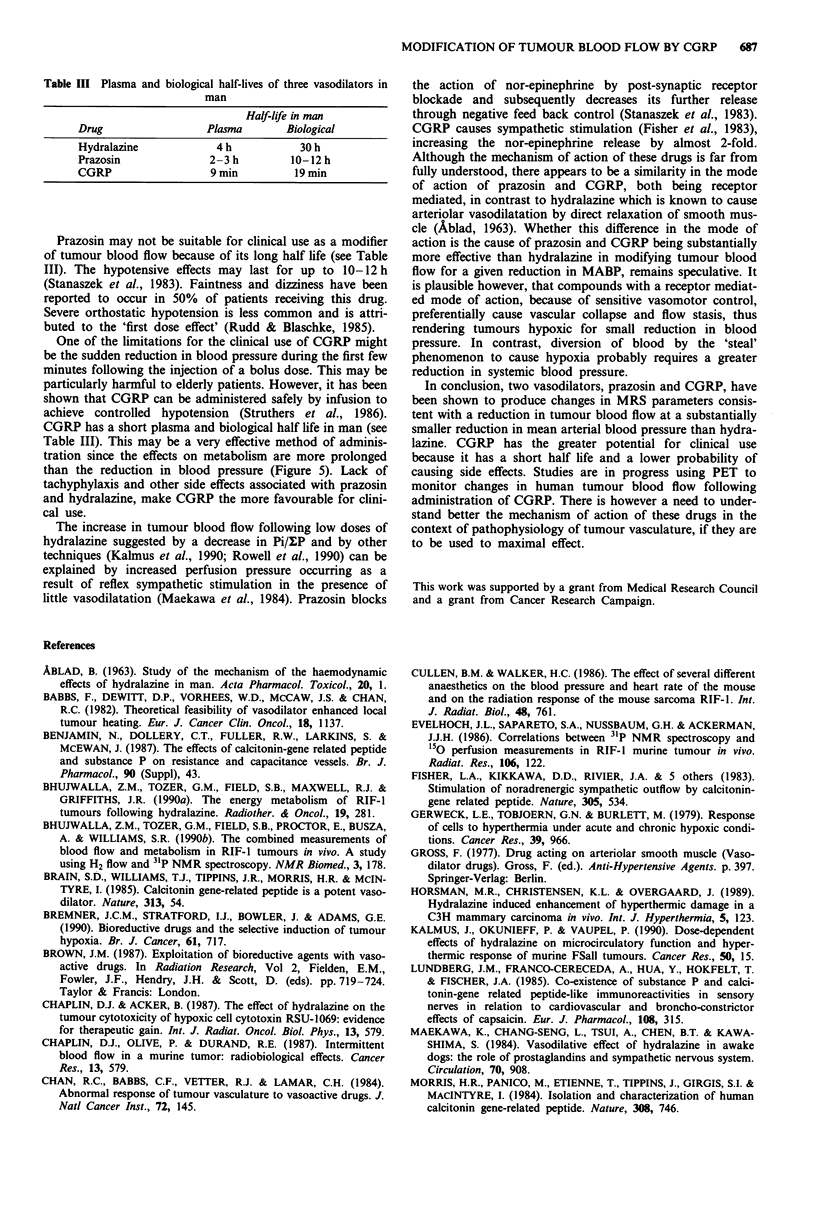

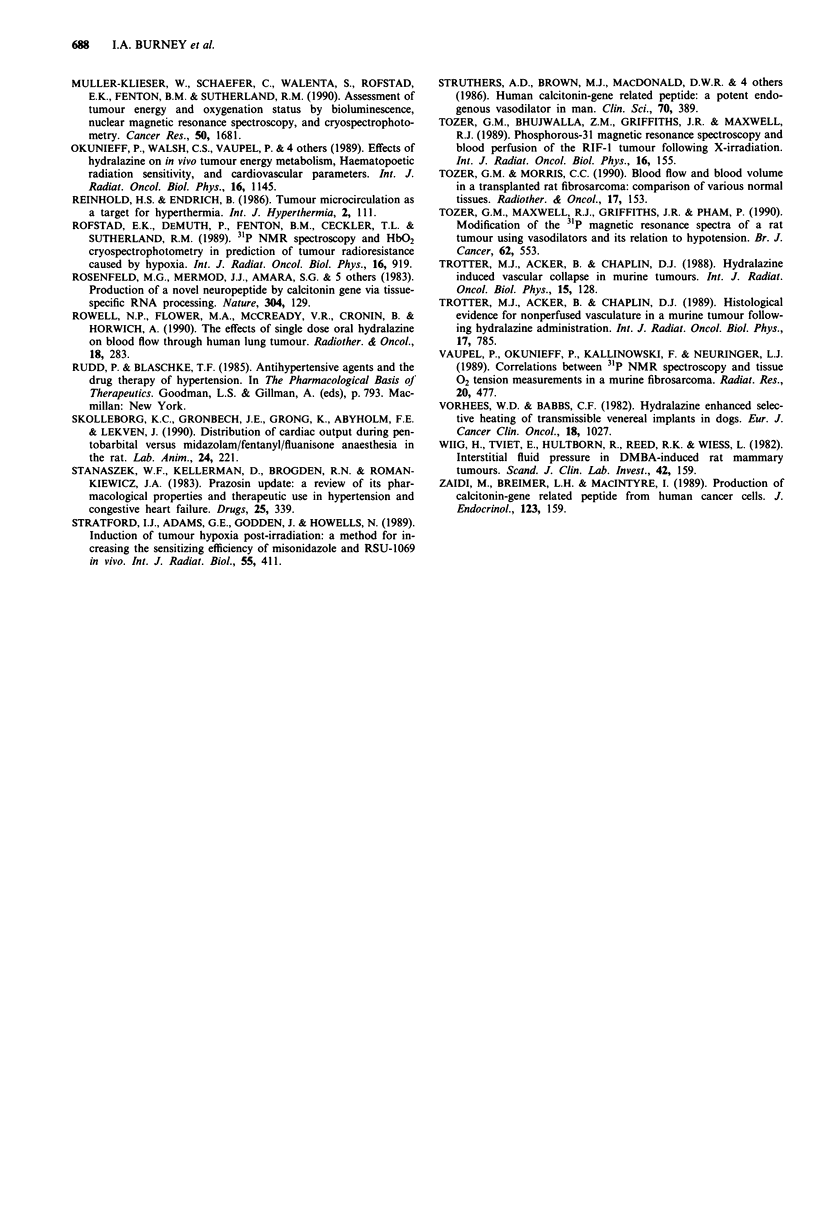


## References

[OCR_00708] ABLAD B. (1963). A study of the mechanism of the hemodynamic effects of hydralazine in man.. Acta Pharmacol Toxicol (Copenh).

[OCR_00711] Babbs C. F., DeWitt D. P., Voorhees W. D., McCaw J. S., Chan R. C. (1982). Theoretical feasibility of vasodilator-enhanced local tumor heating.. Eur J Cancer Clin Oncol.

[OCR_00722] Bhujwalla Z. M., Tozer G. M., Field S. B., Maxwell R. J., Griffiths J. R. (1990). The energy metabolism of RIF-1 tumours following hydralazine.. Radiother Oncol.

[OCR_00727] Bhujwalla Z. M., Tozer G. M., Field S. B., Proctor E., Busza A., Williams S. R. (1990). The combined measurement of blood flow and metabolism in RIF-1 tumours in vivo. A study using H2 flow and 31P NMR spectroscopy.. NMR Biomed.

[OCR_00734] Brain S. D., Williams T. J., Tippins J. R., Morris H. R., MacIntyre I. (1985). Calcitonin gene-related peptide is a potent vasodilator.. Nature.

[OCR_00737] Bremner J. C., Stratford I. J., Bowler J., Adams G. E. (1990). Bioreductive drugs and the selective induction of tumour hypoxia.. Br J Cancer.

[OCR_00757] Chan R. C., Babbs C. F., Vetter R. J., Lamar C. H. (1984). Abnormal response of tumor vasculature to vasoactive drugs.. J Natl Cancer Inst.

[OCR_00748] Chaplin D. J., Acker B. (1987). The effect of hydralazine on the tumor cytotoxicity of the hypoxic cell cytotoxin RSU-1069: evidence for therapeutic gain.. Int J Radiat Oncol Biol Phys.

[OCR_00762] Cullen B. M., Walker H. C. (1985). The effect of several different anaesthetics on the blood pressure and heart rate of the mouse and on the radiation response of the mouse sarcoma RIF-1.. Int J Radiat Biol Relat Stud Phys Chem Med.

[OCR_00716] Dart A. M., Riemersma R. A., Schömig A., Ungar A. (1987). Metabolic requirements for release of endogenous noradrenaline during myocardial ischaemia and anoxia.. Br J Pharmacol.

[OCR_00768] Evelhoch J. L., Sapareto S. A., Nussbaum G. H., Ackerman J. J. (1986). Correlations between 31P NMR spectroscopy and 15O perfusion measurements in the RIF-1 murine tumor in vivo.. Radiat Res.

[OCR_00774] Fisher L. A., Kikkawa D. O., Rivier J. E., Amara S. G., Evans R. M., Rosenfeld M. G., Vale W. W., Brown M. R. (1983). Stimulation of noradrenergic sympathetic outflow by calcitonin gene-related peptide.. Nature.

[OCR_00779] Gerweck L. E., Nygaard T. G., Burlett M. (1979). Response of cells to hyperthermia under acute and chronic hypoxic conditions.. Cancer Res.

[OCR_00789] Horsman M. R., Christensen K. L., Overgaard J. (1989). Hydralazine-induced enhancement of hyperthermic damage in a C3H mammary carcinoma in vivo.. Int J Hyperthermia.

[OCR_00793] Kalmus J., Okunieff P., Vaupel P. (1990). Dose-dependent effects of hydralazine on microcirculatory function and hyperthermic response of murine FSall tumors.. Cancer Res.

[OCR_00797] Lundberg J. M., Franco-Cereceda A., Hua X., Hökfelt T., Fischer J. A. (1985). Co-existence of substance P and calcitonin gene-related peptide-like immunoreactivities in sensory nerves in relation to cardiovascular and bronchoconstrictor effects of capsaicin.. Eur J Pharmacol.

[OCR_00806] Maekawa K., Liang C. S., Tsui A., Chen B. T., Kawashima S. (1984). Vasodilative effect of hydralazine in awake dogs: the roles of prostaglandins and the sympathetic nervous system.. Circulation.

[OCR_00810] Morris H. R., Panico M., Etienne T., Tippins J., Girgis S. I., MacIntyre I. (1984). Isolation and characterization of human calcitonin gene-related peptide.. Nature.

[OCR_00817] Mueller-Klieser W., Schaefer C., Walenta S., Rofstad E. K., Fenton B. M., Sutherland R. M. (1990). Assessment of tumor energy and oxygenation status by bioluminescence, nuclear magnetic resonance spectroscopy, and cryospectrophotometry.. Cancer Res.

[OCR_00824] Okunieff P., Walsh C. S., Vaupel P., Kallinowski F., Hitzig B. M., Neuringer L. J., Suit H. D. (1989). Effects of hydralazine on in vivo tumor energy metabolism, hematopoietic radiation sensitivity, and cardiovascular parameters.. Int J Radiat Oncol Biol Phys.

[OCR_00830] Reinhold H. S., Endrich B. (1986). Tumour microcirculation as a target for hyperthermia.. Int J Hyperthermia.

[OCR_00834] Rofstad E. K., DeMuth P., Fenton B. M., Ceckler T. L., Sutherland R. M. (1989). 31P NMR spectroscopy and HbO2 cryospectrophotometry in prediction of tumor radioresistance caused by hypoxia.. Int J Radiat Oncol Biol Phys.

[OCR_00839] Rosenfeld M. G., Mermod J. J., Amara S. G., Swanson L. W., Sawchenko P. E., Rivier J., Vale W. W., Evans R. M. (1983). Production of a novel neuropeptide encoded by the calcitonin gene via tissue-specific RNA processing.. Nature.

[OCR_00844] Rowell N. P., Flower M. A., McCready V. R., Cronin B., Horwich A. (1990). The effects of single dose oral hydralazine on blood flow through human lung tumours.. Radiother Oncol.

[OCR_00856] Skolleborg K. C., Grönbech J. E., Grong K., Abyholm F. E., Lekven J. (1990). Distribution of cardiac output during pentobarbital versus midazolam/fentanyl/fluanisone anaesthesia in the rat.. Lab Anim.

[OCR_00864] Stanaszek W. F., Kellerman D., Brogden R. N., Romankiewicz J. A. (1983). Prazosin update. A review of its pharmacological properties and therapeutic use in hypertension and congestive heart failure.. Drugs.

[OCR_00868] Stratford I. J., Adams G. E., Godden J., Howells N. (1989). Induction of tumour hypoxia post-irradiation: a method for increasing the sensitizing efficiency of misonidazole and RSU 1069 in vivo.. Int J Radiat Biol.

[OCR_00874] Struthers A. D., Brown M. J., Macdonald D. W., Beacham J. L., Stevenson J. C., Morris H. R., MacIntyre I. (1986). Human calcitonin gene related peptide: a potent endogenous vasodilator in man.. Clin Sci (Lond).

[OCR_00879] Tozer G. M., Bhujwalla Z. M., Griffiths J. R., Maxwell R. J. (1989). Phosphorus-31 magnetic resonance spectroscopy and blood perfusion of the RIF-1 tumor following X-irradiation.. Int J Radiat Oncol Biol Phys.

[OCR_00890] Tozer G. M., Maxwell R. J., Griffiths J. R., Pham P. (1990). Modification of the 31P magnetic resonance spectra of a rat tumour using vasodilators and its relationship to hypotension.. Br J Cancer.

[OCR_00885] Tozer G. M., Morris C. C. (1990). Blood flow and blood volume in a transplanted rat fibrosarcoma: comparison with various normal tissues.. Radiother Oncol.

[OCR_00901] Trotter M. J., Acker B. D., Chaplin D. J. (1989). Histological evidence for nonperfused vasculature in a murine tumor following hydralazine administration.. Int J Radiat Oncol Biol Phys.

[OCR_00907] Vaupel P., Okunieff P., Kallinowski F., Neuringer L. J. (1989). Correlations between 31P-NMR spectroscopy and tissue O2 tension measurements in a murine fibrosarcoma.. Radiat Res.

[OCR_00913] Voorhees W. D., Babbs C. F. (1982). Hydralazine-enhanced selective heating of transmissible venereal tumor implants in dogs.. Eur J Cancer Clin Oncol.

[OCR_00918] Wiig H., Tveit E., Hultborn R., Reed R. K., Weiss L. (1982). Interstitial fluid pressure in DMBA-induced rat mammary tumours.. Scand J Clin Lab Invest.

[OCR_00923] Zaidi M., Breimer L. H., MacIntyre I. (1989). Production of calcitonin gene-related peptide from human cancer cells.. J Endocrinol.

